# Epidemiologic features and clinical course of COVID-19: a retrospective analysis of 19 patients in Germany

**DOI:** 10.2217/fvl-2020-0256

**Published:** 2021-02-24

**Authors:** Sara Elgamasy, Eman Elsayed Sakr, Mohamed Gomaa Kamel, Sherief Ghozy, Ghadeer Gamal Elsayed, Mostafa Mahmoud Fahmy, Ahmed Ghazy, Mohamed Maged Nagaty Ibrahim, Mostafa Ebraheem Morra

**Affiliations:** 1 ^1^Department of Internal Medicine, Klinikum Altmühlfranken Weißenburg, Bayern 91781, Germany; 2 ^2^Cardiology Department, El-Mataria Teaching Hospital, Cairo, Egypt; 3 ^3^Faculty of Medicine, Minia University, Minia 61519, Egypt; 4 ^4^Faculty of Medicine, Mansoura University, Mansoura 35516, Egypt; 5 ^5^Faculty of Medicine, Tanta University, Tanta 31524, Egypt; 6 ^6^Faculty of Medicine, Benha University, Benha 13741, Egypt; 7 ^7^Bariatric Medicine Department, Hamad Medical Corporation, Doha, Qatar; 8 ^8^Faculty of Medicine, AlAzhar University, Cairo 11884, Egypt

**Keywords:** coronavirus, COVID-19, Germany, infection, outbreak, pandemic, PCR, respiratory, retrospective, SARS-CoV-2

## Abstract

**Background:** SARS-coronavirus-2 causes coronavirus disease-19 (COVID-19). **Materials & methods:** We here report epidemiology; clinical, radiological and laboratory characteristics; and outcomes of COVID-19 in 19 patients confirmed by reverse-transcriptase–PCR. **Results:** In 19 PCR-confirmed cases (median age 69 years; 63% males), the most common presentations were fever (79%), cough (79%) and fatigue (79%). The most common comorbidities were hypertension (47%), hypothyroidism (32%) and cardiac diseases (32%). All patients received symptomatic treatment. Ampicillin/sulbactam was prescribed for 50% of cases. Also, 13 (68.4%) recovered and discharged, 9 (47.3%) needed intensive care unit admission and 4 (21.1%) cases died **Conclusion:** The included cases had variable clinical outcomes following supportive and antibiotic treatments. These findings may contribute to development of more effective strategies for infection control.

The recent SARS coronavirus 2 (SARS-CoV-2) outbreak is the third novel CoV within the last 17 years. Most patients present with mild symptoms such as fever, cough, expectoration, headache, myalgia or fatigue or dyspnea [1,2]. Some patients report gastrointestinal manifestations that precede respiratory illness and fever by 1–2 days. However, other patients may develop lethal complications such as pneumonia, acute respiratory distress syndrome, RNAemia, neurological manifestations and multi-organ failure [3]. It is apparent that SARS-CoV-2 is now much more hazardous than expected compared with previous outbreaks such as the H1N1 pandemic which caused 12,429 deaths over a year while SARS-CoV-2 caused more than 13,000 over 5 weeks in the USA [4]. Moreover, the influenza pandemic that infected a third of world’s population and resulted in 50 million deaths in 1918 had a basic reproductive number of 1.8 only while SARS-CoV-2 has an estimated basic reproductive number (R0) of >2–3 [5,6].

Unlike other viral infections such as SARS, SARS-CoV-2 is highly contagious with a relatively long asymptomatic period. Moreover, there is evidence of presymptomatic transmission of the virus [7]. Given the similarity in viral load between symptomatic and asymptomatic patients [8], controlling SARS-CoV-2 transmission is highly challenging. This retrospective analysis reports the epidemiologic features, clinical characteristics, radiological and laboratory findings and outcomes of 19 patients with PCR-confirmed SARS-CoV-2 infection in Germany.

## Materials & methods

### Study design

This is a retrospective analysis of medical records, data from patients with confirmed SARS-CoV-2 in Klinikum Altmühlfranken Weißenburg Hospital, Germany from 1 March 2020 to 31 March 2020, were reviewed. All the methods were performed per the relevant approved guidelines, regulations and declaration of Helsinki. According to the German Medical Association Professional Code of Conduct in Bavaria, ethical approval was not necessary due to the retrospective nature of our investigation and the complete anonymity of reported data (2020–1096).

### Definition & data collection

Patients with laboratory-confirmed SARS-CoV-2 using reverse-transcriptase–PCR (RT–PCR) assay of a specimen collected on a nasopharyngeal swab or throat swab regardless of age and gender of the patient were included. A standardized data extraction form was utilized to collect the data from the medical records. The study mentions no identifying information from the participants and all responses were recorded anonymously. The treating doctor in Klinikum altmühlfranken Weißenburg Hospital is the first author of this research and she provided the medical records to the research group who extracted the data from the records. The extraction form entailed epidemiologic data such as the setting of contact and affected family members. We also reported demographic data such as age, gender and associated comorbidities. Information on clinical symptoms or signs at presentation and x-ray findings were also reported. Moreover, we collected the laboratory markers such as leukocytes and lymphocytes count, red blood cells and hemoglobin. Biochemical variables including lactate dehydrogenase (LDH), alanine aminotransferase (ALT), aspartate aminotransferase (AST), urea, creatinine, C-reactive protein (CRP) and electrolytes were reported. Troponin, procalcitonin and creatine kinase were also collected. Patients were categorized as either needed mechanical ventilation or not.

### Statistical analysis

The data were analyzed by a senior author (SG). All data were analyzed using R software version 4.0.0 using the package ‘Rcmdr’, and graphically presented using packages ‘*ggplot2’*, ‘*ggrepel’* and ‘*ggpubr’*. Median and range were used to represent continuous variables while we used frequencies and percentages to represent categorical variables. To compare patients based on the need for supplemental oxygen, Chi-square/Fisher’s exact tests were used for categorical variables and t-test/Mann–Whitney tests were used for continuous, variables based upon the distribution of the data (normally distributed or not). A p-value < 0.05 was considered significant for all statistical tests.

## Results

### Epidemiologic & clinical features

**Figure 1.**
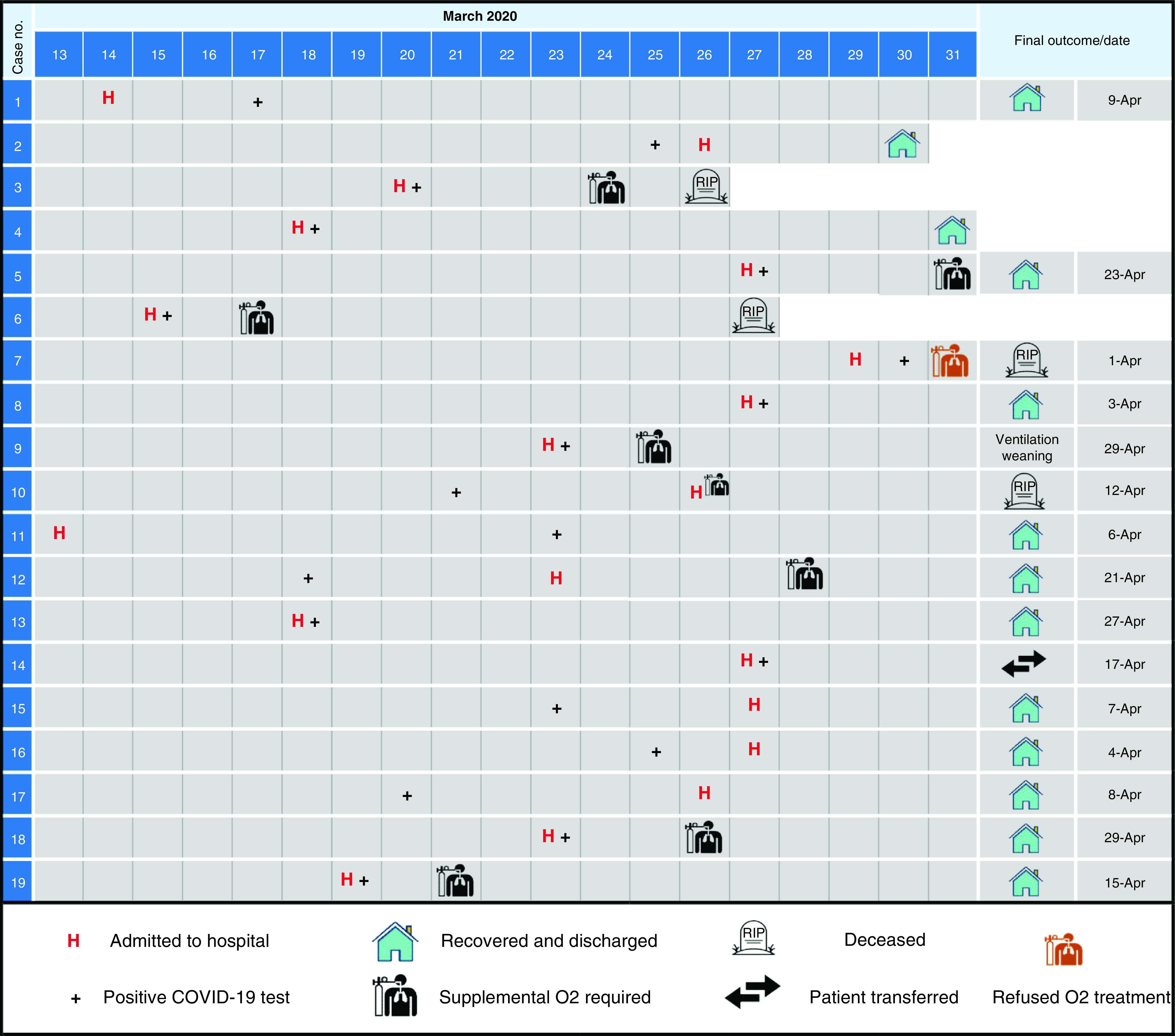
The timeline and outcomes of the 19 included patients.

Between 1 March 2020 and 31 March 2020, a total of 19 cases with PCR-confirmed COVID-19 were managed in the hospital. The timeline of analyzed cases is shown in Figure 1. Of the 19 patients, eight and 11 were assorted into needed mechanical ventilation or not, respectively. The median age of patients was 69 years (range 45 to 90); of them, 12 (63%) were males. Nine patients (47%) had a history of known household contact. Fever (79%), cough (79%), fatigue (79%) and dyspnea (42%) were the most common presentations. The median temperature was 39°C (37–40). Gastrointestinal manifestations were observed in 32% of cases. Three patients had a loss of taste and/or appetite. The median duration from symptom onset to hospital admission was 10 (2–28) days. Most of the subjects had associated comorbidities (89%); hypertension (47%), hypothyroidism (on levothyroxine) (32%) and cardiac problems (32%) were the most common reported comorbidities. Having a COVID-19 positive family member was the only significant risk factor (p = 0.020) for requiring a supplemental oxygen, while all other patients' baseline characteristics were comparable (Table 1).

**Table 1.** Baseline characteristics of patients infected with SARS-CoV-2.

Variables	All patients (n = 19)	Did not require supplemental O2 (n = 11)	Required supplemental O2 (n = 8)	p-value
**Demographics**				
Age, median (range) y	69 (45–90)	82 (53–90)	66 (45–88)	0.129
Male sex, n (%)	12 (63)	6 (55)	6 (75)	0.633
Any comorbidity, n (%)	17 (89)	10 (91)	7 (88)	1.000
History of contact with a SARS-CoV-2 patient	9 (47)	3 (27)	6 (75)	0.150
Positive family member	10 (53)	3 (27)	7 (88)	0.020^§^
**Days from symptoms onset to hospital presentation, median (range)**	10 (2–28)	7 (2–28)	10 (5–21)	0.133
**Signs and symptoms on presentation, n (%)**				
Fever	15 (79)	8 (73)	7 (88)	0.603
Cough	15 (79)	7 (64)	8 (100)	0.103
Fatigue	13 (68)	8 (73)	5 (63)	1.000
Dyspnea/shortness of breath	8 (42)	5 (45)	3 (38)	1.000
GIT manifestations^†^	6 (32)	5 (45)	1 (13)	0.177
Poor general condition	6 (32)	4 (36)	2 (25)	1.000
Dehydration	6 (32)	5 (45)	1 (13)	0.177
Rhinorrhea	5 (26)	4 (36)	1 (13)	0.338
Sputum production	4 (21)	3 (27)	1 (13)	0.603
Sore throat	3 (16)	3 (27)	0 (0)	0.228
Loss of taste and/or appetite	3 (16)	2 (18)	1 (13)	1.000
Headache	2 (11)	1 (9)	1 (13)	1.000
Myalgia	1 (5)	1 (9)	0 (0)	1.000
**Comorbidities, n (%)**				
HTN	9 (47)	5 (45)	4 (50)	1.000
Hypothyroidism	6 (32)	5 (45)	1 (13)	0.177
Cardiac conditions^‡^	6 (32)	4 (36)	2 (25)	1.000
Type 2 diabetes mellitus	3 (16)	2 (18)	1 (13)	1.000
BPH	3 (16)	2 (18)	1 (13)	1.000
Obesity	2 (11)	0 (0)	2 (25)	0.164
Other chronic conditions	8 (42)	4 (36)	4 (50)	0.658
**Vital signs at presentation, median (range)**				
Temperature,°C	39 (37–40)	38 (38–39)	39 (37–40)	0.130
Heart rate,/min	88 (63–116)	88 (63–116)	88 (67–116)	1.000
Systolic blood pressure, mm Hg	142 (100–175)	153 (100–175)	128 (117–163)	0.091
Pulse oximeter O_2_ saturation, %	96 (78–100)	93 (78–100)	97 (86–100)	0.904

† Includes abdominal pain, nausea, vomiting and diarrhea;

‡ Includes atrial fibrillation, chronic heart disease/failure, A–V block and coronary artery disease,

§ Statistically significant.

### Imaging & laboratory findings

Baseline auscultation revealed abnormal chest in 14 (74%) of cases; 12 (63%) had bilateral rales or crepitations while two (11%) had bilateral wheezes. Radiologically, 15 patients (79%) had abnormal chest x-rays: 12 with bilateral chest infiltrates and two with unilateral infiltrates.

Median total leukocytes count was equal in patients who required O_2_ supplementation (n = 11, 58%) to those who did not require O_2_. Besides, the median glomerular filtration rate was 62 ml/min/1.73 m^2^. Serum urea, creatinine, AST, ALT and total bilirubin revealed normal to elevated values; 39 mg/dl (12–129 mg/dl) and 1 mg/dl (0.6–2.2 mg/dl), 24 U/l (9–117 U/l), 50 U/l (16–147 U/l) and 0.5 mg/dl (0.2–1.6 mg/dl), respectively. The same pattern was observed for procalcitoni.

DM: diabetes mellitus; n 0.12 ng/ml (0.02–0.64 ng/ml) in 15 cases. LDH and CRP were elevated in all patients at baseline investigations; 343 U/l (169–931 U/l) and 8 mg/dl (1–17 mg/dl), respectively. Creatine kinase was reported in seven cases; with 1115 and 561 U/l in two cases and normal range in the remaining five cases. Troponin was assessed in nine cases at baseline 13.09 (90.72–4.75) pg/ml. Moreover, ferritin levels were evaluated in seven patients, all of them revealed elevated values with an overall median of 824 (287–3429) ng/ml. Furthermore, the median segmented neutrophils and lymphocytes values for 15 cases were 72 and 15%, respectively. Median values for sodium, potassium, chloride and calcium showed slight to no elevation, and they were 133, 3.9, 96.5 and 2.2 mmol/l, respectively. Among patients requiring supplemental oxygen, the ALT (p = 0.016) and AST (p = 0.026) levels were significantly higher as compared with those who did not (Table 2).

**Table 2.** Baseline clinical findings of patients infected with SARS-CoV-2.

Variables	All patients (n = 19)	Did not require supplemental O2 (n = 11)	Required supplemental O2 (n = 8)	p-value
**Baseline investigations, median (range)**				
TLC, ×10^9^/l	6 (4–10)	6 (4–10)	6 (4–9)	0.778
RBC, ×10^6^/l	5 (4–5)	5 (4–5)	4 (4–5)	0.310
Hemoglobin, g/dl	13 (11–17)	14 (11–17)	13 (12–15)	0.177
Segmented neutrophils, %	72 (57–88)	68 (57–88)	75 (66–84)	0.694
Lymphocytes, %	15 (2–28)	18 (2–28)	11 (3–21)	0.397
Platelets, ×10^9^/l	216 (85–487)	208 (121–415)	241 (85–487)	0.657
ALT (U/l)	24 (9–117)	19 (9–46)	40.5 (20–117)	0.016^†^
AST (U/l)	50 (16–147)	30 (16–86)	61.5 (30–147)	0.026^†^
Total bilirubin (mg/dl)	0.5 (0.2–1.6)	0.5 (0.3–1.6)	0.5 (0.2–1.1)	0.968
GFR (CKD-EPI), ml/min/1.73 m^2^	62 (28–117)	54 (28–103)	93 (32–117)	0.051
Urea (mg/dl)	39 (12–129)	35 (20–129)	41.5 (12–82)	0.904
Serum Creatinine (mg/dl)	1 (0.6–2.2)	1.1 (0.6–2.2)	0.8 (0.7–1.9)	0.177
LDH, U/l	343 (169–931)	299 (169–675)	357 (265–931)	0.146
C-reactive protein, mg/l	8 (1–17)	6 (1–17)	12 (3–17)	0.075
Procalcitonin (ng/ml)	0.12 (0.02–0.64)	0.12 (0.02–0.64)	0.12 (0.07–0.38)	0.694
K^+^ (mEq/l)	3.9 (3.1–5.1)	3.8 (3.1–5.1)	4 (3.4–4.9)	0.840
Na^+^ (mEq/l)	133 (128–143)	133 (128–143)	132.5 (130–137)	0.545
Cl^−^(mEq/l)	96.5 (91–102)	96.5 (91–101)	97 (91–102)	0.897
Ca^2+^ (mEq/l)	2.2 (1.6–2.5)	2.3 (1.6–2.5)	2.1 (2.1–2.3)	0.177
**Baseline chest radiograph, n (%)**				
Abnormal chest radiograph	15 (79)	8 (73)	7 (88)	0.603
Bilateral lung infiltrates	12 (63)	5 (45)	7 (88)	0.147
Unilateral lung infiltrates	2 (11)	2 (18)	0 (0)	0.485
**Baseline auscultation findings, n (%)**				
Abnormal auscultation	14 (74)	9 (82)	5 (63)	0.603
Bilateral rales/crepitation	12 (63)	7 (64)	5 (63)	1.000
Bilateral wheezes	2 (11)	2 (18)	0 (0)	0.485

† Statistically significant.

GFR: Glomerular filtration rate; LDH: Lactate dehydrogenase; RBC: Red blood cell; TLC: Total leukocyte count.

**Figure 2.**
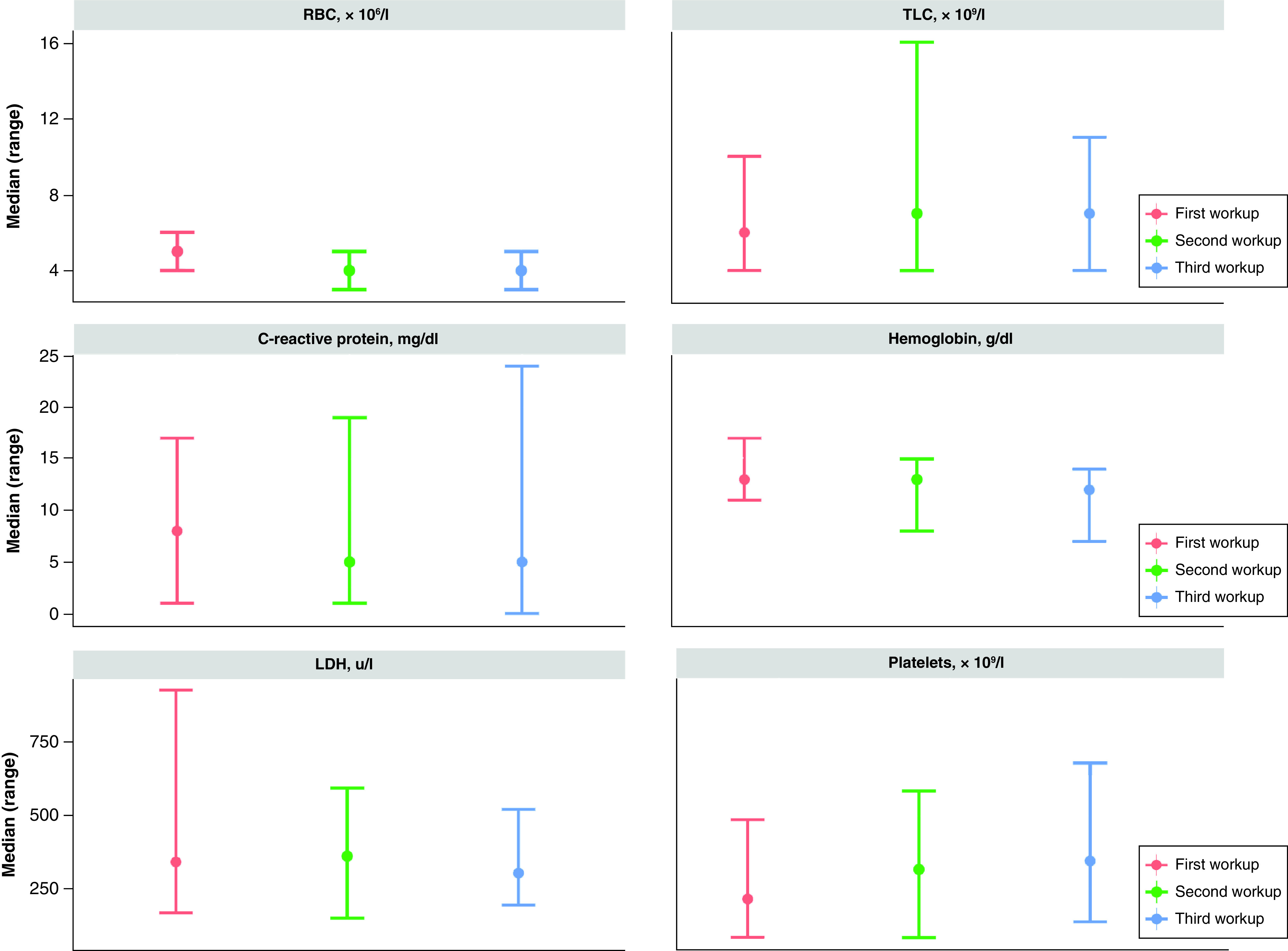
Laboratory workups for the 19 included patients. Not all patients had all laboratory values assessed at second and third workup. LDH: Lactate dehydrogenase; RBC: Red blood cell; TLC: Total leukocyte count.

Figure 2 displays the median and range for three laboratory workups. The median (range) for TLC was 6 × 10^9^/l (4–10 × 10^9^/l), 7 × 10^9^/l (4–16 × 10^9^/l) and 7 × 10^9^/l (4–11 × 10^9^/l) for first, second and third workups, respectively. CRP reached its highest value on admission workup 8 mg/dl (1–17 mg/dl), then it decreased to 5 mg/dl for both the second and third workups. Whereas LDH was higher in the second 363 U/l (151–596 U/l) than in the first 343 U/l (169–931 U/l) and third 305 U/l (196–523 U/l) workups. Median hemoglobin concentrations were higher in the first (13 g/dl [11–17 g/dl]) and second (13 g/dl [8–15 g/dl]) workups as opposed to the third workup (12 g/dl [7–14 g/dl]). The highest median platelet count was recorded in the third workup (346 × 10^9^/l [138–681 × 10^9^/l]); whereas it measured 216 × 10^9^/l (85–487 × 10^9^/l) and 317 × 10^9^/l (84–585 × 10^9^/l) in the first and second workups, respectively.

### Outcomes

All patients received symptomatic treatment including normal saline and antipyretics. Of 18 patients who received antibiotics, ampicillin/sulbactam was prescribed for 50%. Out of 19 cases, 13 (68.4%) recovered and discharged, 9 (47.3%) needed intensive care unit admission and 4 (21.1%) cases died (Table 3).

**Table 3.** Management and outcomes of the patients.

Case	Treatment		Outcome
	Antibiotics	Symptomatic treatment	
1	Ampicillin/sulbactam	Infusions/paracetamol (1 gm) iv.	Recovered and discharged
2	None	Paracetamol, analgesics, saline	Recovered and discharged
3	Ampicillin/sulbactam 3 gm TDS	2 l saline, paracetamol iv. infusion, metamizole, antitussive, secretolytic, sometimes paracodine, 8–10 l of oxygen	Died (DNR)
4	Cefpodoxime/initial referral from family physician with suspected choledocholithiasis	Antiemetic (MCP), probiotics, normal saline, O_2_ 2 l and ambroxol	Recovered and discharged
5	Cirpofloxacine by the family doctor but stopped in the hospital at 27 March)	Saline, paracetamol, antitussive, antipyretics, secretolytics, euthyroxin 75 mg once daily	Recovered and discharged
6	Ampicillin/sulbactam 3 g TD	Tamiflu 75 mg/BD, perfalgan, ambroxol, salbutamol, atrovent, MCP as needed, O_2_ 10 l, paracodeine, hydroxychloroquin	Died
7	Ampicillin/sulbactam 3 g TD	Saline, paracetamol 1 gm, N-acetyl cystine, ambroxol, urapidil 12.5 mg when needed, morphine	Died (DNR)
8	Ampicillin/sulbactam and cefpodoxime given by family physician for 1 week before admission and then stopped for abdominal pain	Normal saline, paracetamol, acetylcysteine, ambroxol, O_2_, L-thyroxin, candesartan	Recovered and discharged
9	Ciprofloxacin by family physician, piperacillin-tazobactam and roxithromycin	Paracetamol, acetylcysteine, ambroxol, O_2_, potassium, novaminsulfon (novalgin), normal saline, clopidogrel, atorvastatin, ramipril but stopped later because of the angiotensin receptor blockers theory)	Sent for weaning
10	(Piperacillin/tazobactam)	O_2_	Died
11	1. Ampicillin/sulbactam/suspicion of recurrent urosepsis. 2. Meropenem: +ve for *Pseudomonas aeruginosa* on urine culture with resistance for ampicillin-sulbactam.	1. Paracetamol, normal saline, acetylcysteine, novalgin 2. Other ttt: ramipril, L-thyroxin, sitagliptin, levodopa, cabidopa, rapid insulin, toujeo, benserazide, pantoprazole	Recovered and discharged
12	Ampicillin/sulbactam, piperacillin tazobactam	Paracetamol, iv. saline, salbutamol, atrovent INH, pregabalin, bisoprolol	Recovered and discharged
13	Amoxi/clav 2.2 g	Paracetamol, normal saline, ASA, LMWH, acetylcysteine, ambroxol, betahistine, novaminsulfon, metoprolol, tamsulosin, mirtazipine, simvastatin	Recovered and discharged
14	Ampicillin/sulbactam 3 g TD	Normal saline, paracetamol, Amproxol, acetylcysteine, novaminsulfon, probiotics, pntoprazol	Transferred to another hospital for cardiac catheterization due to suspected CHD
15	Ceftriaxone	Paracetamol, acetylcysteine, ambroxol, O_2_ 2 l, novaminsulfon (novalgin), normal saline	Recovered and discharged
16	Co-amoxi/clav	iv. saline, 1–2 l O_2_	Recovered and discharged
17	Amoxi/clav 2.2 g/high CRP	O_2_ 2 l, perfalgan, jonosteril (fluid and electrolyte replacement), dimenhydrinate/vomex (when needed), paracetamol/metamizole	Recovered and discharged
18	Ampicillin/sulbactam/piperacillin sulbactam/hydroxychloroquine	Acetylcysteine/cortisol/perfalgan – 6 l O_2_	Recovered and discharged
19	Piperacillin/tazobactam (high CRP)/meropenem (high CRP despite being on piperacillin/tazobactam)	Perfalgan, novalgin, pantoprazol, O_2_, ambroxol, MCP, morphin, heparin, laxan, renal dialysis (24/03–08/04), erythrocyte concentrate (anemic)	Recovered and discharged

CHD: Coronary heart disease; DNR: A do-not-resuscitate order; iv.: Intravenous; MCP: Metoclopramide; TDS: Three-times a day.

## Discussion

In this study, we report detailed epidemiologic and clinical features of 19 patients with PCR-confirmed SARS-CoV-2 infection in Germany. The most common symptoms at the onset of COVID-19 were fever, fatigue and cough. The median duration from symptom onset to hospital presentation was 10 (2–28) days. This duration was longer in patients who required supplemental O_2_ (10 [5–21 days]) compared with patients who did not require O_2_ (7 [2–28 days]). Nine (47%) patients had a history of known household contact. In contrast to a previous study from Beijing, the ratio of severe cases of the COVID-19 infection was 42% in our study while they had 17.6% only [9]. Moreover, 42% of our patients required O_2_ supplementation and 21% died. Although this finding should be interpreted cautiously due to the small sample, a previous study in Italy on 1591 patients reported that 81.7% of them needed respiratory support where 88% needed invasive endotracheal intubation and 11% need noninvasive mechanical ventilation as well as 13% needed oxygen mask. Besides, they showed an intensive care unit mortality rate of 26% [10]. Other previous studies demonstrated that 26–33% of patients might require intensive care [1,11,12] while 76–90% of patients require supplemental oxygen [1,12,13] and they had 4–15% mortality rate [1,11,12].

32% of our cases presented with co-existing gastrointestinal symptoms (abdominal pain, nausea, vomiting or diarrhoea). Indeed, gastrointestinal symptoms have been reported in 2–10% of SARS-CoV-2 patients in Wuhan, the origin of the infection. Moreover, they occurred 1–2 days before the development of respiratory illness (dyspnea and fever, for example) [1]. Zhang *et al.* have explained this finding after detecting angiotensin-converting enzyme-II receptors, the gate of SARS-CoV-2 into host cells, throughout ileum and colon [14]. Accordingly, SARS-CoV-2 virus can potentially be transmitted through both the respiratory as well as gastrointestinal routes.

It is noteworthy as well that hypertension was the most common comorbidity among our patients (47%). Despite our small sample size, this finding agrees with a large study on 5700 patients where hypertension co-existed in 56.6% of positive cases [15]. The latter reported obesity and diabetes as the second and third most common comorbidities with rates of 41.7 and 33.8%, respectively. Whereas our patients revealed hypothyroidism and cardiac diseases in the second position (Six patients; 32%); and diabetes and benign prostatic hyperplasia equally in the third position (three patients; 16%). Further analysis is required to provide optimum protection and screening facilities to patients at risk.

In our analysis, among the abnormalities aforestated, most patients showed lymphopenia and elevated AST as well as ALT, whereas all patients revealed high CRP and LDH. This is consistent with a recent study that reported a high-accuracy association between abnormal laboratory findings and being positive for SARS-CoV-2 to the extent that they can even predict the PCR results [16].

Moving on to diagnosis, diagnosing SAR-CoV-2 based on PCR testing is challenging due to the high rate of false-negative results [17], hence the importance of radiology. CT is the radiological investigation of choice in SARS-CoV-2 [18], but chest radiograph was beneficial in our cases. Of note, the chest radiograph is considered insensitive early in the course of illness, and it is a screening tool in hospitals with limited resources; it can show abnormalities late in the disease. However, the chest x-ray revealed abnormal lung infiltrates in 15 (79%) patients. This is consistent with Wong and colleagues, who studied chest x-ray findings in 64 patients and reported baseline abnormalities in 51 (79.7%) patients in the form of consolidations and ground glass opacities [19]. Hence, the cost–effectiveness of chest radiographs warrants further research for determining its discriminatory power. Accordingly, health authorities should repeat the PCR, isolate suspected cases, use CT (due to having a higher accuracy) and test as many as possible of suspected cases otherwise infected cases could undetectably transmit the virus. Our study has been limited by small sample size, this is because Klinikum Altmühlfranken Weißenburg is a small hospital and the study was conducted earlier in the outbreak of COVID-19 in Germany.

## Conclusion

With the tremendous measures in administering newly synthesized COVID-19 vaccines, the current treatment protocols are depending mainly on supportive treatment and trying previously used drugs due to our experience on the posology, safety profile, side effects, as well as drug interactions of these drugs which were used before for SARS- and MERS-CoVs infections [20], since they share some similar genetic and clinical characteristics [21,22].

Among the 19 patients diagnosed with SARS-CoV-2 infection in Germany, we show that the clinical presentation was frequently mild respiratory tract infections. Yet, some patients required supplemental oxygen and had variable clinical outcomes following treatment with supportive and antibiotic treatment. These findings may contribute to the development of more effective strategies of SARS-CoV-2 control.

Indeed, the infection continues to spread in around 213 countries and territories with the confirmed cases and deaths exceeded the total numbers of infected and died patients with SARS and MERS-CoVs [20]. Yet, the real numbers of cases remain undoubtedly much higher due to limited screening and testing. In contrast, the announced relatively low numbers in low and middle-income countries could be attributed to their strict protocols of fewer PCR tests because of limited resources, whereas the Infectious Diseases Society of America has suggested a prioritization of testing. The priority is given mainly to symptomatic healthcare staff, immunocompromised, elderly and critically ill patients with severe respiratory distress with no obvious reason. Besides, according to the ‘iceberg’ theory, some countries (the UK) test severe cases only while others test for mild cases as well (Germany), which resulted in a much lower mortality rate between both countries (14 vs 4.7%, respectively). Moreover, these high numbers of infection may be partially attributed to late lockdown and screening since many of these greatly affected countries have big cities and airports for tourists from all over the world. With the very short incubation period and false-negative PCR, long-term and international control of COVID-19 could be challenging to achieve [23].

**Summary points**

- SARS-coronavirus-2 causes coronavirus disease-19 (COVID-19).
- This study is detailing epidemiologic and clinical features of 19 patients with PCR-confirmed SARS-CoV-2 infection in Germany.
- The most common symptoms at the onset of COVID-19 were fever, fatigue and cough.
- The median duration from symptom onset to hospital presentation was 10 (2–28) days.
- These findings may contribute to development of more effective strategies for infection control.
